# An animal model study on the gene expression profile of meniscal degeneration

**DOI:** 10.1038/s41598-020-78349-4

**Published:** 2020-12-08

**Authors:** Yehan Fang, Hui Huang, Gang Zhou, Qinghua Wang, Feng Gao, Chunbao Li, Yujie Liu, Jianping Lin

**Affiliations:** 1 Medical School of Chinese PLA and Chinese PLA General Hospital, Beijing, China; 2 Department of Orthopedic Surgery, Hainan General Hospital (Hainan Affiliated Hospital of Hainan Medical University), Haikou, Hainan China; 3 Department of Nursing, Hainan General Hospital (Hainan Affiliated Hospital of Hainan Medical University), Haikou, Hainan China; 4 Department of Sports Injury and Arthroscopy Surgery, National Institute of Sports Medicine, Beijing, China

**Keywords:** Molecular biology, Medical research, Molecular medicine

## Abstract

Meniscal degeneration is a very common condition in elderly individuals, but the underlying mechanisms of its occurrence are not completely clear. This study examines the molecular mechanisms of meniscal degeneration. The anterior cruciate ligament (ACL) and lateral collateral ligament (LCL) of the right rear limbs of seven Wuzhishan mini-pigs were resected (meniscal degeneration group), and the left rear legs were sham-operated (control group). After 6 months, samples were taken for gene chip analysis, including differentially expressed gene (DEG) analysis, gene ontology (GO) analysis, clustering analysis, and pathway analysis. The selected 12 DEGs were validated by real time reverse transcription-polymerase chain reaction (RT-PCR). The two groups showed specific and highly clustered DEGs. A total of 893 DEGs were found, in which 537 are upregulated, and 356 are downregulated. The GO analysis showed that the significantly affected biological processes include nitric oxide metabolic process, male sex differentiation, and mesenchymal morphogenesis, the significantly affected cellular components include the endoplasmic reticulum membrane, and the significantly affected molecular functions include transition metal ion binding and iron ion binding. The pathway analysis showed that the significantly affected pathways include type II diabetes mellitus, inflammatory mediator regulation of TRP channels, and AMPK signaling pathway. The results of RT-PCR indicate that the microarray data accurately reflects the gene expression patterns. These findings indicate that several molecular mechanisms are involved in the development of meniscal degeneration, thus improving our understanding of meniscal degeneration and provide molecular therapeutic targets in the future.

## Introduction

Meniscal degeneration is characterized by abnormal meniscus signals and extrusions at imaging, and it most often occurs in middle-aged and elderly patients with osteoarthritis. Meniscal degeneration has been regarded as one of the characteristics of osteoarthritis, but recent studies support that the normal meniscus has a significant protective effect on the articular cartilage^1,2^. Meniscal degeneration leads to loss of meniscal function, causing early-stage cartilage degeneration and subchondral bone loss, and leading to osteoarthritis and poor quality of life^3,4^. Therefore, delaying meniscal degeneration is considered important for preventing and treating osteoarthritis, but the specific molecular mechanisms for meniscal degeneration are not completely clear.

Due to ethics issues and limited donor menisci, especially normal control menisci, it is nearly impossible to conduct research on meniscal degeneration directly in humans, and appropriate animal models are required. Previous studies have often used small animals such as guinea pigs, rats, and rabbits for conducting research on meniscal degeneration^5–9^. Unfortunately, the animals themselves and their knee joints and menisci are small, and the results can hardly be translated to humans when considering the characteristics of the human knee joint weight-bearing and meniscal degeneration. The knee joint of mini-pigs has weight-bearing characteristics closer to that of humans.

Therefore, this study aims to examine the molecular mechanisms of meniscal degeneration. Based on the Pond-Nuki model of anterior cruciate ligament (ACL) resection^10^, the present study established models of meniscal degeneration using ACL and lateral collateral ligament (LCL) resection, and the gene chip analysis technology was used to study the changes in early meniscus degeneration gene expression profiles, providing evidence for studying the possible mechanism and treatment of meniscal degeneration.

## Materials and methods

### Pig model of meniscal degeneration

Seven Wuzhishan mini-pigs (Institute of Animal Science and Veterinary Medicine, Hainan Academy of Agricultural Sciences), with a mean age of 6.8 ± 0.3 (range: 6.1–7.4) months and a mean body weight (BW) of 19.4 ± 3.4 (range: 18.6–21.2) kg, were used as experimental animals. Only male pigs were included in the current study to avoid the variability induced by sexual hormones. All animals were anesthetized by intramuscular injection of xylazine hydrochloride (0.3 ml/kg BW, Shengda Animal Medicine Co., Ltd., Shengda, China) combined with pentobarbital sodium (20 mg/kg BW, Merck Serono KGaA, Darmstadt, Germany).

After the surgical sites were shaved and sterilized, the right rear limbs (as the Ba group) were operated at approximately 10.0 cm from the patella to the tibial tubercle by using a lateral parapatellar approach. The joint was opened partly by medial patella luxation. The ACL was fixed by a clamp and was cut by 1.0 cm at the distal end, as described previously^11^. The LCL was separated and exposed along the joint line and resected for a length of 1.0 cm. After washing with 1 00 ml of sterile saline, the articular capsule was sutured intermittently and independently with 3-0 silk sutures (Aipu Medical Equipment Co., Ltd., Hangzhou, China). The operation was carried out on the left rear limbs as the sham group (Aa group) as similar to the above incision of the articular capsule, but no manipulation was made to the ligaments, menisci, cartilage, or bone. There was no randomization regarding the grouping of the rear limbs to allow for easy recognition of the experimental and control limbs after the operation.

The mini-pigs were checked twice a day after the operation. All animals were treated with penicillin (Kerui Animal Pharmaceutical Co., Ltd, Chengdu, China) and tramadol (Duoduo Pharmaceutical Co., Ltd, Jiamusi, China) for 7 days to avoid infections. After the wound was completely healed, the animal could move freely in the pigpen.

### Preparation procedure of meniscal tissue

The animals were sacrificed at 26 weeks after surgery by acute massive exsanguination. The menisci were isolated using a standardized preparation technique. After the knee joint was opened from the front, the joint capsule and the ligaments were cut along the joint line near the side of the femur to separate the tibia from the femur. The medial menisci were carefully removed in one piece from the tibia. They were washed with phosphate-buffered saline (PBS) and cut into soybean-sized pieces. The preparation was carried out without touching the surfaces of the menisci or the hyaline articular cartilage to prevent contamination and destruction of the meniscal surface and deep structures. The samples were taken from the red-white zone of the meniscal pars intermedia, followed by placing in a cryopreservation tube for immediate freezing and storing in liquid nitrogen. The sampling was performed immediately after the death of the mini-pigs and was completed within 10 min. All equipment was treated with hydrogen peroxide to remove eventual RNAse.

### RNA extraction and assessment

After DNase digestion, the total RNA from the meniscal tissue was extracted using an E.Z.N.A Total RNA Kit II (Omega Bio-Tek Inc., Norcross, GA, USA) according to the manufacturer’s instructions. The quantity and quality of RNA were measured using a NanoDrop ND-1000 spectrophotometer (Thermo Fisher Scientific, Waltham, MA, USA). RNA integrity was assessed by standard denaturing agarose gel electrophoresis.

### Agilent microarray study

RNA labeling and array hybridization were performed according to the Agilent One-Color Microarray-Based Gene Expression Analysis protocol (Agilent Technologies, Santa Clara, CA, USA). Briefly, the total RNA from each sample was linearly amplified and labeled with Cy3-UTP using the Agilent Quick Amp One-Color labeling kit (Agilent Technologies, Santa Clara, CA, USA). The Labeled cRNAs were purified using the RNeasy Mini Kit (Qiagen, Venlo, The Netherlands) according to the manufacturer’s instructions. The concentration and specific activity of the labeled cRNAs (pmol Cy3/μg cRNA) were measured using a NanoDrop ND-1000 spectrophotometer (Thermo Fisher Scientific, Waltham, MA, USA). After that, 1 µg of each labeled cRNA was fragmented by adding 11 μl of 10 × blocking agent and 2.2 μl of 25 × fragmentation buffer and heating the mixture at 60 °C for 30 min. Finally, 55 μl of 2 × GE hybridization buffer was added to dilute the labeled cRNA. Then, 100 μl of the hybridization solution was dispensed into the gasket slide and assembled to the gene expression microarray slide. The slides were incubated for 17 h at 65 °C in a hybridization oven (Agilent Technologies, Santa Clara, CA, USA). The RNA was hybridized to the Whole Pig Genome Microarray (Agilent Technologies, Santa Clara, CA, USA). The hybridized arrays were washed, fixed, and scanned using a DNA Microarray Scanner (G2505C, Agilent Technologies, Santa Clara, CA, USA)^12^.

### Microarray data analyses

The raw gene expression data were extracted from the array images using the Feature Extraction software (version 11.0.1.1, Agilent Technologies, Santa Clara, CA, USA). Quantile normalization and subsequent data processing were performed with the GeneSpring GX v12.1 software package (Agilent Technologies, Santa Clara, CA, USA). After quantile normalization of the raw data, the genes from at least seven out of the 14 samples had flags detected (“All Targets Value”) and were chosen for further data analysis. Statistically significant differentially expressed genes (DEGs) between the two groups were identified through volcano plot filtering, using a threshold of fold-change > 2.0 and P-value < 0.05 based on the Student t-test in the GeneSpring software). DEGs between the two samples were identified through fold change filtering. Unsupervised hierarchical clustering was performed using the R scripts to compare gene expression profiles among the samples of the same group. Gene ontology (GO, http://geneontology.org) analysis and pathway analysis were performed with the standard enrichment computation method to associate the DEGs with GO categories and pathways. The GO categories comprised of three structured networks (biological processes, cellular components, and molecular function) of defined terms to describe the gene functions. To perform the GO analysis of differential genes, top GO was used to infer the molecular function they are involved in^12^. The KEGG pathway analysis was performed to infer the pathways the DEGs are involved in^13–15^. All the microarray data are MIAME compliant, and the raw data are available through the GEO database with the accession number GSE145402.

### Real-time RT-PCR analysis

Twelve genes were selected for validation. The quantification of transcript levels for the selected genes and the housekeeping gene GAPDH was performed using the ViiA 7 Real-time PCR System (Applied Biosystems, Foster City, CA, USA). PCR primers were designed based on cDNA sequences from the NCBI Sequence database using Primer 5.0 (PREMIER Biosoft International, Palo Alto, CA, USA). The Gene Amp PCR System 9700 (Applied Biosystems, Foster City, CA, USA) was used to generate the first-strand cDNA from the isolated RNA. The cDNA was amplified with an initial Taq DNA polymerase activation step at 95 °C for 10 min, followed by 40 cycles of denaturation at 95 °C for 10 s and annealing at 60 °C for 60 s, according to the manufacturer’s instructions. Each reaction was repeated three times, and the Ct values were obtained in triplicates for each gene. The expression level of the selected genes was normalized to GAPDH using the 2^−ΔΔCt^ method. Each real-time RT-PCR experiment was repeated twice in triplicate. Statistical significance of the differences between the two groups was determined by the Student t-test while P < 0.05, using SPSS 19.0 for Windows (IBM, Armonk, NY, USA).

### Ethical approval

All procedures were reviewed and approved by the Medical Ethics Committee of Hainan General Hospital (Med-Eth-Re [2020] 5). All experiments were performed in accordance with the relevant guidelines and regulations.

## Results

### DEG identification

**Figure 1:**
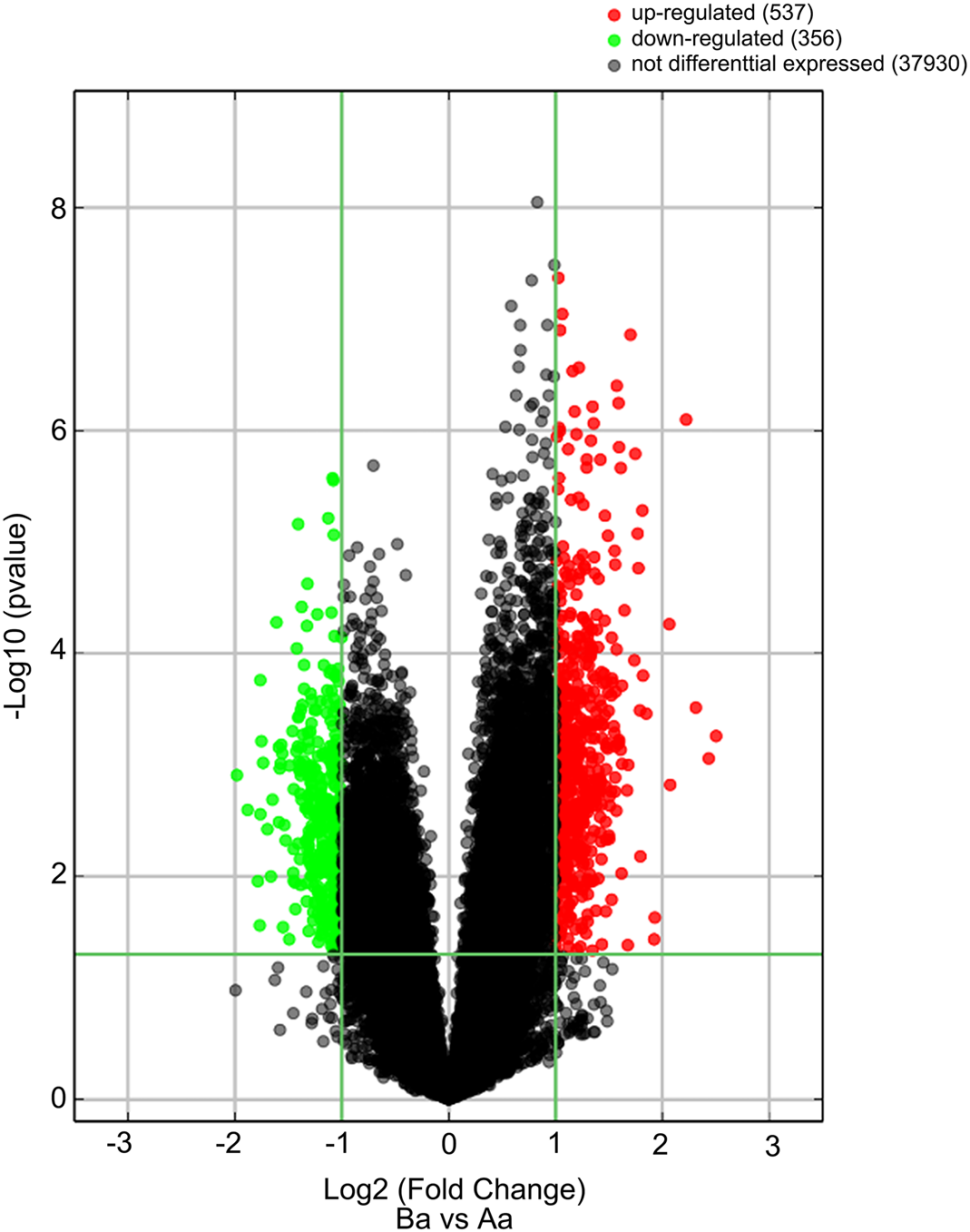
The volcano plot map of all the genes. Differentially expressed genes (DEGs) were determined while fold-change > 2 and P-value < 0.05. 893 DEGs were found, among which 537 are upregulated and 356 are down-regulated.

The volcano map of all the data obtained after the chip scan can easily and reasonably reflect the distribution of the DEGs between the two groups (Fig. 1). According to the DEG criteria of fold-change > 2 and P-value < 0.05, 893 DEGs were found, among which 537 are upregulated and 356 are down-regulated. The top ten upregulated DEGs include TFAP2D, HOXD13, CES1, PAK5, C7H6orf15, GCNT7, VIL1, COL7A1, CD81, and SLC7A8 (Table 1). The top ten down-regulated DEGs include SPMI, INA, CPS1, PGA5, LYRM4, TMCO5A, LOC100520832, CFAP58, ESRP1, and VMA21 (Table 2).

**Table 1:** The top ten differential genes with up-regulation.

Genbank accession	Gene symbol	Description	P-value	FDR	Fold change
XM_003356639	*TFAP2D*	Transcription factor AP-2 delta	0.000551587	0.023345329	5.6602732
XM_003483680	*HOXD13*	Homeobox D13	0.000877072	0.028532155	5.3957126
NM_214246	*CES1*	Sus scrofa carboxylesterase 1 (CES1)	5.4688E-05	0.008853756	4.1784836
AK393241	*PAK5*	Rep: Serine/threonine-protein kinase PAK 7—Mus musculus (Mouse), partial (21%)	1.72044E-05	0.00514212	3.4211194
NM_001128447	*C7H6orf15*	Sus scrofa chromosome 7 open reading frame, human C6orf15 (C7H6orf15)	8.41952E-06	0.003760236	3.4049223
NM_001113699	*GCNT7*	Sus scrofa glucosaminyl (N-acetyl) transferase family member 7 (GCNT7)	1.38326E-07	0.000537464	3.2506906
AK231343	*VIL1*	Rep: cyclic peptide transporter precursor—Methylobacteriumextorquens PA1, partial (4%)	0.001001336	0.030467417	3.2032195
XM_005669519	*COL7A1*	Collagen type VII alpha 1 chain	0.000982785	0.030115232	3.0775177
BX670110	*CD81*	Sus Scrofa library (scac) Sus scrofa cDNA clone scac0034.n.11 3prim	1.4119E-06	0.001443671	3.0181273
XM_003128550	*SLC7A8*	Solute carrier family 7 member 8	7.2232E-05	0.010036037	2.8769457

**Table 2:** The top ten differential genes with down-regulation.

Genbank accession	Gene symbol	Description	P-value	FDR	Fold change
NM_001031776	*SPMI*	Sus scrofa seminal plasma sperm motility inhibitor/spermadhesin AQN-3-like protein (SPMI)	0.002536463	0.047702946	3.6789604
XM_001929320	*INA*	Internexin neuronal intermediate filament protein alpha	0.002054744	0.042969359	3.1323546
AK394043	*CPS1*	Carbamoyl-phosphate synthase 1	5.26505E-05	0.008631794	3.0512209
NM_213873	*PGA5*	Sus scrofa pepsinogen 5, group I (pepsinogen A) (PGA5)	0.003462281	0.056295579	2.9000267
AK233279	*LYRM4*	Ribonuclease P/MRP subunit p40	0.036770923	0.197230012	2.8101604
XM_001927899	*TMCO5A*	Transmembrane and coiled-coil domains 5A	0.009299658	0.095443941	2.7383115
NM_001285972	*LOC100520832*	Sus scrofalithostathine-like (LOC100520832)	0.005710985	0.073428296	2.7296819
XM_005671443	*CFAP58*	Cilia and flagella associated protein 58	0.019700007	0.140371132	2.6994439
AK396999	*ESRP1*	Epithelial splicing regulatory protein 1	0.000755832	0.026771077	2.6832393
AK343969	*VMA21*	VMA21, vacuolar ATPase assembly factor	0.000363673	0.01979061	2.6355477

### Unsupervised hierarchical clustering

**Figure 2:**
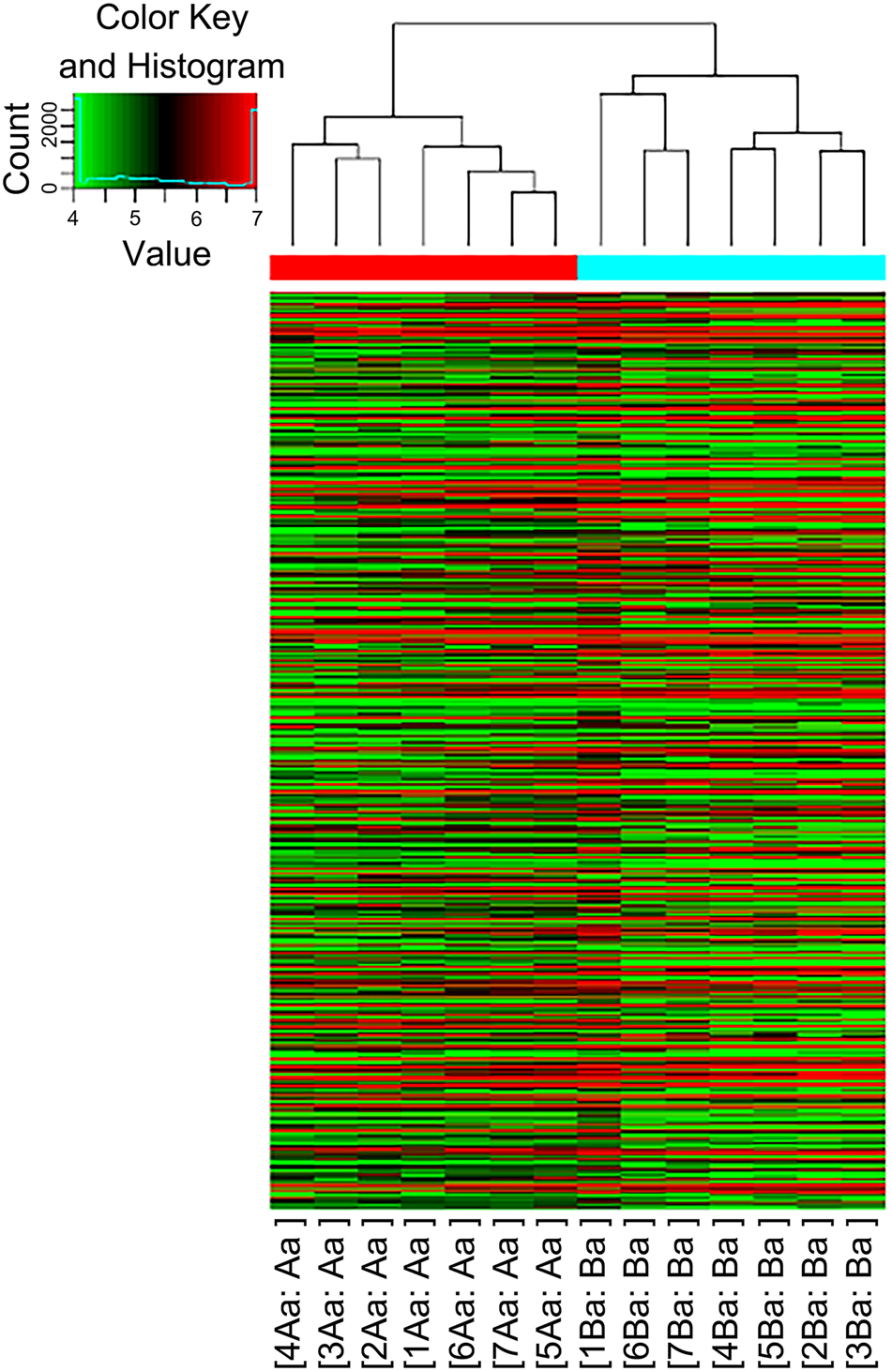
The dendrogram. The meniscal degeneration group is shown in cyan blue, while the control group is shown in red.

The unsupervised cluster analysis of DEGs obtained after comparison of the two groups demonstrate the relationship as well as the difference between the groups. The results show that the two groups are well separated in terms of the DEGs (Fig. 2). All samples are accurately clustered into the same cluster of the corresponding category, indicating the reliability of the results of chip analysis, and that the gene expression pattern of the experimental group in comparison to the control group is consistent.

### Results of the GO enrichment analysis

**Figure 3:**
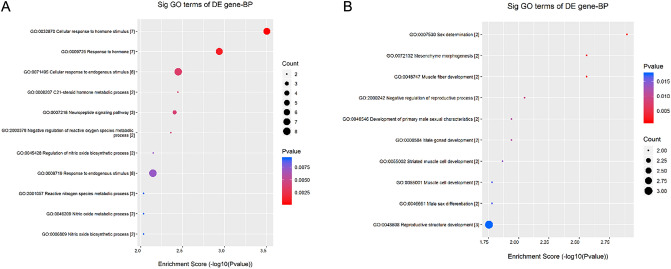
The biological processes enrichment analyses for the differentially expressed genes (DEGs) analysed by Gene Ontology (GO). (**A**) The top ten biological processes with upregulated Enrichment Score Dot Plot. (**B**) The top ten biological processes with down-regulated Enrichment Score Dot Plot.

The DEGs obtained from the above analysis were classified into biological processes, cellular components, and molecular functions according to the functional relevance of the genes. The top ten biological processes with upregulated gene enrichment scores included the cellular response to hormone stimulus, response to hormones, cellular response to endogenous stimulus, C21-steroid hormone metabolic process, neuropeptide signaling pathway, negative regulation of reactive oxygen species metabolic process, regulation of nitric oxide biosynthetic process, response to endogenous stimulus, nitric oxide biosynthetic process, and nitric oxide metabolic process (Table 3 and Fig. 3A).

**Table 3:** The biological processes with up-regulated gene enrichment scores.

GO.ID	Term	P value	Enrichment_Score	Genes
GO:0032870	Cellular response to hormone stimulus	0.00031193	3.505944124	*NR5A1//SLC2A4//ADIPOR2//SOCS2//P2RY4//MYOD1//HCRTR2*
GO:0009725	Response to hormone	0.00115203	2.938536525	*NR5A1//SLC2A4//HCRTR2//ADIPOR2//SOCS2//P2RY4//MYOD1*
GO:0071495	Cellular response to endogenous stimulus	0.00356694	2.447703824	*NR5A1//SLC2A4//HCRTR2//ADIPOR2//SOCS2//P2RY4//MYOD1//FGF21*
GO:0008207	C21-steroid hormone metabolic process	0.00358808	2.445137806	*CYP17A1//PRL*
GO:0007218	Neuropeptide signaling pathway	0.00394089	2.404406092	*SCG5//NPY2R//HCRTR2*
GO:2000378	Negative regulation of reactive oxygen species metabolic process	0.00435963	2.360550537	*PRL//ACP5*
GO:0045428	Regulation of nitric oxide biosynthetic process	0.00708678	2.149550997	*PRL//ACP5*
GO:0009719	Response to endogenous stimulus	0.00714517	2.14598759	*NR5A1//SLC2A4//HCRTR2//ADIPOR2//SOCS2//P2RY4//MYOD1//FGF21*
GO:0006809	Nitric oxide biosynthetic process	0.00923586	2.034522585	*PRL//ACP5*
GO:0046209	Nitric oxide metabolic process	0.00923586	2.034522585	*PRL//ACP5*
GO:2001057	Reactive nitrogen species metabolic process	0.00923586	2.034522585	*PRL//ACP5*
GO:0048732	Gland development	0.01028441	1.987820429	*PRL//CSN2//NR5A1//HOXD13*
GO:0042445	Hormone metabolic process	0.01130856	1.946592525	*CYP17A1//PRL//NR5A1*
GO:1903426	Regulation of reactive oxygen species biosynthetic process	0.01163811	1.934117409	*PRL//ACP5*
GO:0007589	Body fluid secretion	0.01293115	1.888362813	*PRL//CSN2*
GO:0042446	Hormone biosynthetic process	0.01569514	1.804234938	*CYP17A1//PRL*
GO:0034754	Cellular hormone metabolic process	0.01868863	1.728422521	*CYP17A1//PRL*
GO:1903409	Reactive oxygen species biosynthetic process	0.02026862	1.693175919	*PRL//ACP5*
GO:0042136	Neurotransmitter biosynthetic process	0.02190261	1.65950407	*PRL//ACP5*
GO:0007631	Feeding behavior	0.02358953	1.627280649	*NPY2R//HCRTR2*
GO:0042221	Response to chemical	0.02361703	1.626774706	*PTK2B//S100A12//CCR10//NR5A1//SOCS2//SLC52A2//ACP5//SLC2A4//HCRTR2//ADIPOR2//P2RY4//MYOD1//PRL//FGF21*
GO:0070887	Cellular response to chemical stimulus	0.0238929	1.621731065	*PTK2B//S100A12//NR5A1//SOCS2//SLC2A4//HCRTR2//ADIPOR2//P2RY4//MYOD1//PRL//FGF21*
GO:0046849	Bone remodeling	0.02711786	1.566744651	*ACP5//SPP2*
GO:0030879	Mammary gland development	0.03679131	1.434254721	*PRL//CSN2*
GO:1901700	Response to oxygen-containing compound	0.04136472	1.383369859	*ACP5//SLC2A4//SOCS2//P2RY4//MYOD1//HCRTR2*
GO:0009755	Hormone-mediated signaling pathway	0.04314004	1.365119451	*ADIPOR2//NR5A1*
GO:0042133	Neurotransmitter metabolic process	0.04314004	1.365119451	*PRL//ACP5*
GO:2000377	Regulation of reactive oxygen species metabolic process	0.04314004	1.365119451	*PRL//ACP5*
GO:1901652	Response to peptide	0.04836282	1.315488391	*SLC2A4//SOCS2//HCRTR2*

The top ten biological processes with down-regulated gene enrichment scores included sex determination, muscle fiber development, mesenchymal morphogenesis, negative regulation of the reproductive process, male gonad development, primary male sexual characteristics development, striped muscle cell development, male sex differentiation, muscle cell development, and reproductive structure development (Table 4 and Fig. 3B).

**Table 4:** The biological processes with down-regulated gene enrichment scores.

GO.ID	Term	P value	Enrichment_Score	Genes
GO:0007530	Sex determination	0.00128145	2.89230002	*DMRT1//NR5A1*
GO:0048747	Muscle fiber development	0.00275321	2.560160546	*ACTA1//LEF1*
GO:0072132	Mesenchyme morphogenesis	0.00275321	2.560160546	*LEF1//ACTA1*
GO:2000242	Negative regulation of reproductive process	0.00895073	2.048141367	*DMRT1//NR5A1*
GO:0008584	Male gonad development	0.01149866	1.939352658	*DMRT1//NR5A1*
GO:0046546	Development of primary male sexual characteristics	0.01149866	1.939352658	*NR5A1//DMRT1*
GO:0055002	Striated muscle cell development	0.01359466	1.866631736	*ACTA1//LEF1*
GO:0046661	Male sex differentiation	0.01662697	1.779187017	*NR5A1//DMRT1*
GO:0055001	Muscle cell development	0.01662697	1.779187017	*ACTA1//LEF1*
GO:0048608	Reproductive structure development	0.0175686	1.755262805	*NR5A1//DMRT1//LEF1*
GO:0061458	Reproductive system development	0.0179883	1.745009826	*NR5A1//DMRT1//LEF1*
GO:0010817	Regulation of hormone levels	0.01884459	1.724813227	*NR5A1//TTR//SSTR2*
GO:0030855	Epithelial cell differentiation	0.02062492	1.685607828	*DMRT1//LEF1//UPK2*
GO:0042445	Hormone metabolic process	0.02628193	1.580342705	*TTR//NR5A1*
GO:0051146	Striated muscle cell differentiation	0.0333557	1.476829929	*ACTA1//LEF1*
GO:0009888	Tissue development	0.03500311	1.455893416	*LEF1//UPK2//ACTA1//DMRT1//NR5A1*
GO:0008406	Gonad development	0.03550021	1.449769058	*NR5A1//DMRT1*
GO:0045137	Development of primary sexual characteristics	0.03550021	1.449769058	*NR5A1//DMRT1*
GO:0060485	Mesenchymal development	0.03881534	1.410996639	*LEF1//ACTA1*
GO:2000241	Regulation of reproductive process	0.03881534	1.410996639	*DMRT1//NR5A1*
GO:0007517	Muscle organ development	0.04108929	1.386271408	*LEF1//ACTA1*
GO:0010171	Body morphogenesis	0.04880594	1.311527279	*LEF1*
GO:0021879	Forebrain neuron differentiation	0.04880594	1.311527279	*LEF1*
GO:0032673	Regulation of interleukin-4 production	0.04880594	1.311527279	*LEF1*
GO:0045840	Positive regulation of mitotic nuclear division	0.04880594	1.311527279	*DMRT1*
GO:0060713	Labyrinthine layer morphogenesis	0.04880594	1.311527279	*LEF1*

**Figure 4:**
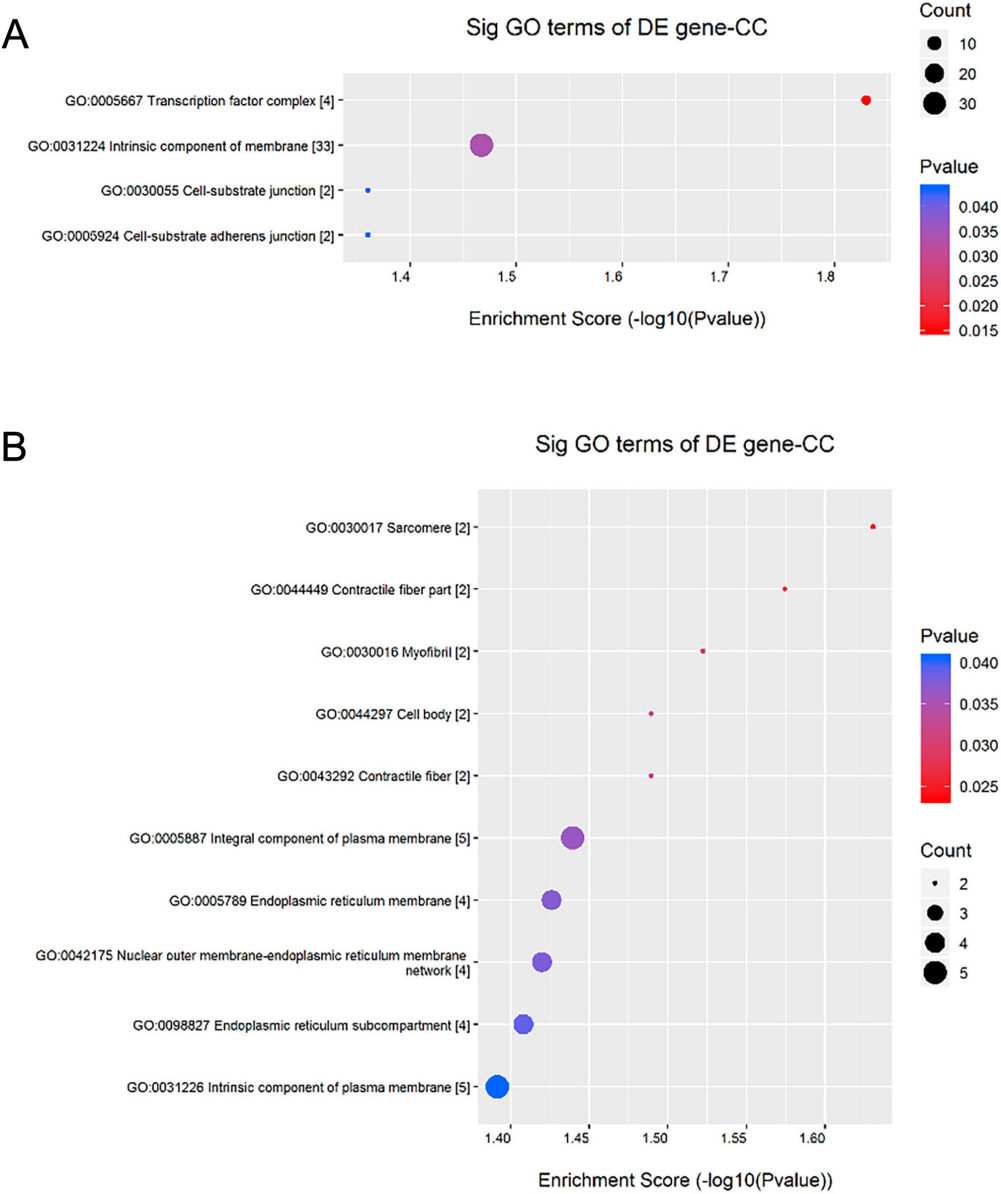
The cellular components enrichment analyses for the differentially expressed genes (DEGs) analysed by Gene Ontology (GO). (**A**) The Cellular components with upregulated Enrichment Score Dot Plot. (**B**) The Cellular components with down-regulated Enrichment Score Dot Plot.

The cellular components involved with upregulated DEGs included transcription factor complex, an intrinsic component of membrane, cell-substrate adherens junction, and cell-substrate junction (Table 5 and Fig. 4A). Cellular components involved with down-regulated DEGs included sarcomere, contractile fiber part, myofibril, contractile fiber, cell body, integral component of the plasma membrane, endoplasmic reticulum membrane, nuclear outer membrane-endoplasmic reticulum membrane network, endoplasmic reticulum subcompartment, intrinsic component of the plasma membrane, plasma membrane part, and endoplasmic reticulum part (Table 6 and Fig. 4B).

**Table 5:** The Cellular components with up-regulated gene enrichment scores.

GO.ID	Term	P value	Enrichment_Score	Genes
GO:0005667	Transcription factor complex	0.01480675	1.829540202	*NR5A1//GTF2B//MYOD1//TFAP2D*
GO:0031224	Intrinsic component of membrane	0.03410257	1.467212853	*SLC2A4//TMPRSS15//IL2RG//IGF2R//NPY2R//CYP2C33//CYP4A21//ADIPOR2//P2RY4//CCR10//CD81//MUC13//GCNT7//GABBR1//ACE2//DLK2//HRK//LOC100156225//ADAM30//IGSF1//CLDN6//SYNDIG1L//LOC100520032//LOC100520992//CWH43//PAQR5//RXFP1//VNN1//HCRTR2//SLC52A2//TSPAN1//SLC25A44//DLG3*
GO:0005924	Cell-substrate adherens junction	0.04360798	1.360434073	*PTK2B//SMPX*
GO:0030055	Cell-substrate junction	0.04360798	1.360434073	*PTK2B//SMPX*

**Table 6:** The Cellular components with down-regulated gene enrichment scores.

GO.ID	Term	P value	Enrichment_Score	Genes
GO:0030017	Sarcomere	0.02341886	1.630434303	*ACTA1//SMPX*
GO:0044449	Contractile fiber part	0.02664338	1.574410611	*SMPX//ACTA1*
GO:0030016	Myofibril	0.03003724	1.522340041	*SMPX//ACTA1*
GO:0043292	Contractile fiber	0.03239074	1.489579145	*SMPX//ACTA1*
GO:0044297	Cell body	0.03239074	1.489579145	*PDYN//ACTA1*
GO:0005887	Integral component of plasma membrane	0.03635516	1.439433886	*SSTR2//SLC12A5//CASR//HTR2C//ACKR2*
GO:0005789	Endoplasmic reticulum membrane	0.03748493	1.426143298	*EPHX1//CYP2E1//RTN4//VMA21*
GO:0042175	Nuclear outer membrane-endoplasmic reticulum membrane network	0.03800801	1.42012482	*EPHX1//CYP2E1//RTN4//VMA21*
GO:0098827	Endoplasmic reticulum subcompartment	0.03906695	1.408190446	*EPHX1//CYP2E1//RTN4//VMA21*
GO:0031226	Intrinsic component of plasma membrane	0.04059138	1.39156617	*SLC12A5//CASR//HTR2C//ACKR2//SSTR2*
GO:0044459	Plasma membrane part	0.04118319	1.385280052	*SLC12A5//CASR//HTR2C//ACKR2//UPK2//SGCZ//SSTR2*
GO:0044432	Endoplasmic reticulum part	0.04936582	1.306573638	*EPHX1//CYP2E1//RTN4//VMA21*

**Figure 5:**
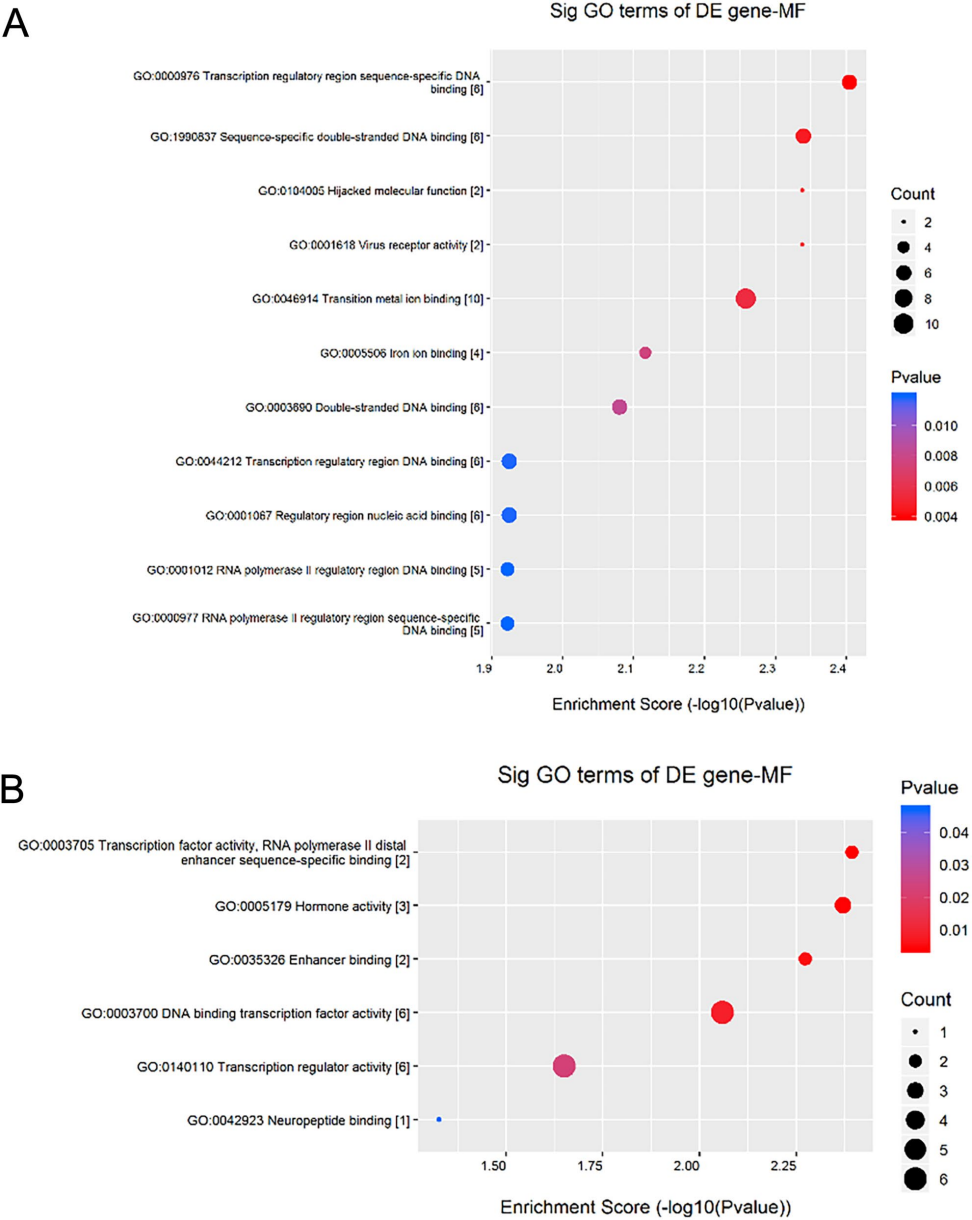
The molecular function enrichment analyses for the differentially expressed genes (DEGs) analysed by Gene Ontology (GO). (**A**) The top ten molecular functions with upregulated Enrichment Score Dot Plot. (**B**) The molecular functions with upregulated Enrichment Score Dot Plot.

The top ten molecular functions with upregulated gene enrichment scores included transcription regulatory region sequence-specific DNA binding, sequence-specific double-stranded DNA binding, virus receptor activity, hijacked molecular function, transition metal ion binding, iron ion binding, double-stranded DNA binding, regulatory region nucleic acid binding, transcription regulatory region DNA binding, and RNA polymerase II regulatory region sequence-specific DNA binding (Table 7 and Fig. 5A). The molecular functions involved with downregulated DEGs included transcription factor activity, RNA polymerase II distal enhancer sequence-specific binding, horizon activity, enhancer-binding, DNA binding transcription factor activity, transcription regulator activity, and neuropeptide binding (Table 8 and Fig. 5B).

**Table 7:** The molecular functions with up-regulated gene enrichment scores.

GO.ID	Term	P value	Enrichment_Score	*GENES*
GO:0000976	Transcription regulatory region sequence-specific DNA binding	0.00394022	2.404479912	*TFAP2D//MYOD1//HOXD13//GTF2B//NR5A1//KLF1*
GO:1990837	Sequence-specific double-stranded DNA binding	0.00457772	2.339350843	*NR5A1//TFAP2D//MYOD1//HOXD13//GTF2B//KLF1*
GO:0001618	Virus receptor activity	0.00458925	2.338258437	*SLC52A2//CLDN6*
GO:0104005	Hijacked molecular function	0.00458925	2.338258437	*SLC52A2//CLDN6*
GO:0046914	Transition metal ion binding	0.00552051	2.258020627	*CYP2C33//CYP4A21//CYP17A1//ACP5//NR5A1//TRIM26//TRIM55//TRIM40//S100A12//SEC23B*
GO:0005506	Iron ion binding	0.00764193	2.116796715	*ACP5//CYP2C33//CYP4A21//CYP17A1*
GO:0003690	Double-stranded DNA binding	0.00832021	2.079865574	*NR5A1//TFAP2D//MYOD1//HOXD13//GTF2B//KLF1*
GO:0001067	Regulatory region nucleic acid binding	0.01189735	1.92454989	*NR5A1//TFAP2D//MYOD1//HOXD13//GTF2B//KLF1*
GO:0044212	Transcription regulatory region DNA binding	0.01189735	1.92454989	*NR5A1//TFAP2D//MYOD1//HOXD13//GTF2B//KLF1*
GO:0000977	RNA polymerase II regulatory region sequence-specific DNA binding	0.01196138	1.922218857	*MYOD1//HOXD13//GTF2B//NR5A1//TFAP2D*
GO:0001012	RNA polymerase II regulatory region DNA binding	0.01196138	1.922218857	*TFAP2D//MYOD1//HOXD13//GTF2B//NR5A1*
GO:0001158	Enhancer sequence-specific DNA binding	0.01223529	1.912385766	*NR5A1//HOXD13*
GO:0004497	Monooxygenase activity	0.01493105	1.82590963	*CYP17A1//CYP4A21//CYP2C33*
GO:0003705	Transcription factor activity, RNA polymerase II distal enhancer sequence-specific binding	0.01501133	1.823580805	*NR5A1//MYOD1*
GO:0046872	Metal ion binding	0.01558956	1.80716619	*LOC106505565//CYP2C33//CYP4A21//CYP17A1//CSN2//TNNC2//KCNIP1//DLK2//S100A12//ACP5//NR5A1//TRIM26//TRIM55//TRIM40//SEC23B//ACE2//GTF2B//ADAM30*
GO:0043169	Cation binding	0.01926006	1.715342436	LOC106505565//CYP2C33//CYP4A21//CYP17A1//CSN2//TNNC2//KCNIP1//DLK2//S100A12//ACP5//NR5A1//TRIM26//TRIM55//TRIM40//SEC23B//ACE2//GTF2B//ADAM30
GO:0035326	Enhancer binding	0.0196297	1.707086296	NR5A1//HOXD13
GO:0004866	Endopeptidase inhibitor activity	0.02517915	1.598959005	ITIH2//COL7A1//CSN2
GO:0061135	Endopeptidase regulator activity	0.02925729	1.533765915	ITIH2//COL7A1//CSN2
GO:0030414	Peptidase inhibitor activity	0.03032845	1.518149723	ITIH2//COL7A1//CSN2
GO:0004857	Enzyme inhibitor activity	0.03165318	1.499582599	SOCS2//ITIH2//COL7A1//CSN2
GO:0001047	Core promoter binding	0.03235762	1.49002349	GTF2B//MYOD1
GO:0001653	Peptide receptor activity	0.03481855	1.458189356	NPY2R//CCR10//HCRTR2
GO:0008528	G-protein coupled peptide receptor activity	0.03481855	1.458189356	NPY2R//CCR10//HCRTR2
GO:0016705	Oxidoreductase activity, acting on paired donors, with incorporation or reduction of molecular oxygen	0.03599219	1.443791785	CYP17A1//CYP4A21//CYP2C33
GO:0020037	Heme binding	0.03599219	1.443791785	CYP2C33//CYP4A21//CYP17A1
GO:0046906	Tetrapyrrole binding	0.03963483	1.401922989	CYP2C33//CYP4A21//CYP17A1
GO:0061134	Peptidase regulator activity	0.04477304	1.348983405	ITIH2//COL7A1//CSN2
GO:0004867	Serine-type endopeptidase inhibitor activity	0.04520724	1.344792035	ITIH2//COL7A1
GO:0043167	Ion binding	0.04984315	1.302394546	LOC106505565//CYP2C33//CYP4A21//CYP17A1//CSN2//TNNC2//KCNIP1//DLK2//S100A12//MYH7//NLRC3//PTK2B//RAB33A//RAB25//NR5A1//ACP5//TRIM26//TRIM55//TRIM40//SEC23B//FASN//ACE2//GTF2B//ADAM30//DAO

**Table 8:** The molecular functions with up-regulated gene enrichment scores.

GO.ID	Term	P value	Enrichment_Score	Genes
GO:0003705	Transcription factor activity, RNA polymerase II distal enhancer sequence-specific binding	0.00403475	2.394183037	*NR5A1//LEF1*
GO:0005179	Hormone activity	0.00426023	2.370566551	*PDYN//TTR//MLN*
GO:0035326	Enhancer binding	0.00532467	2.273707091	*NR5A1//LEF1*
GO:0003700	DNA binding transcription factor activity	0.00872967	2.05900229	*NR5A1//LEF1//DMRT1//ATF4//OTX2//ATF7*
GO:0140110	Transcription regulator activity	0.02236277	1.650474363	*DMRT1//ATF4//OTX2//ATF7//NR5A1//LEF1*
GO:0042923	Neuropeptide binding	0.04714984	1.326519782	*SSTR2*

### Results of the pathway analysis

**Figure 6:**
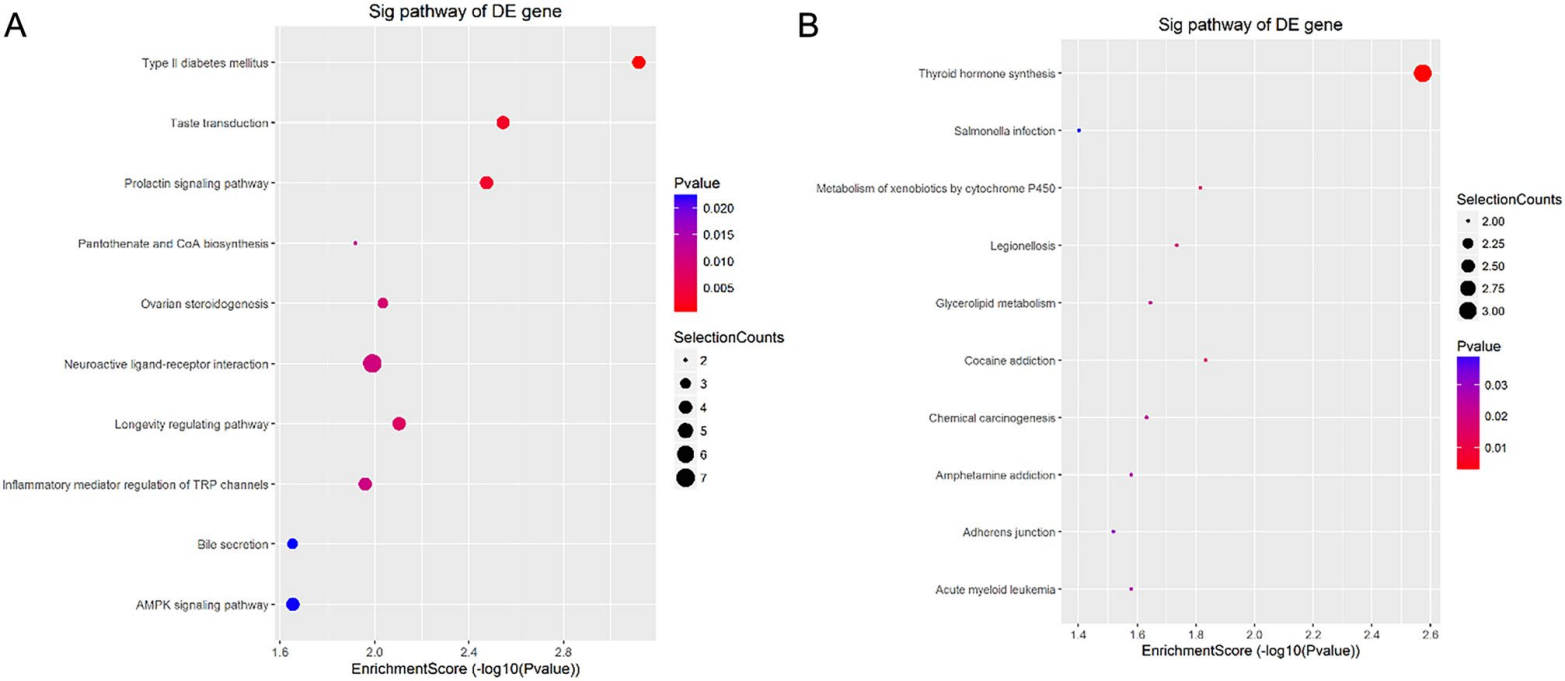
Pathways analyses for the differentially expressed genes (DEGs) analysed by Kyoto Encyclopedia of Genes and Genomes (KEGG). (**A**) The top ten pathways with upregulated Enrichment Score Dot Plot. (**B**) The top ten pathways with down-regulated Enrichment Score Dot Plot.

According to the DEGs, a total of 36 signaling pathways with differential regulation were found. Among these, the top ten pathways included type II diabetes mellitus, taste transduction, prolactin signaling pathway, longevity regulating pathway, ovarian steroidogenesis, neuroactive ligand-receptor interaction, inflammatory mediator regulation of TRP channels, pantothenate and CoA biosynthesis, AMPK signaling pathway, and bile secretion. The down-regulated signaling pathways included thyroid hormone synthesis, cocaine addiction, metabolism of xenobiotics by cytochrome P450, legionellosis, glycerolipid metabolism, chemical carcinogenesis, amphetamine addiction, acute myeloid leukemia, adherens junction, Salmonella infection, protein digestion and absorption, and hematopoietic cell lineage (Tables 9 and 10 and Fig. 6).

**Table 9:** The pathways with up-regulated gene enrichment scores.

Pathway ID	Definition	Fisher-P value	Enrichment_Score	Genes
ssc04930	Type II diabetes mellitus—Sus scrofa (pig)	0.000759595	3.119418	*ABCC8//ADIPOQ//SLC2A4//SOCS2*
ssc04742	Taste transduction—Sus scrofa (pig)	0.002858715	2.543829	*ADCY4//ADCY6//GABBR1//P2RY4*
ssc04917	Prolactin signaling pathway—Sus scrofa (pig)	0.003352503	2.474631	*CSN2//CYP17A1//PRL//SOCS2*
ssc04211	Longevity regulating pathway—Sus scrofa (pig)	0.007875137	2.103742	*ADCY4//ADCY6//ADIPOQ//ADIPOR2*
ssc04913	Ovarian steroidogenesis—Sus scrofa (pig)	0.009215694	2.035472	*ADCY4//ADCY6//CYP17A1*
ssc04080	Neuroactive ligand-receptor interaction—Sus scrofa (pig)	0.01021098	1.990932	*ADRA2C//GABBR1//HCRTR2//NPY2R//P2RY4//PRL//RXFP1*
ssc04750	Inflammatory mediator regulation of TRP channels—Sus scrofa (pig)	0.01098087	1.959363	*ADCY4//ADCY6//CYP2C33//TRPA1*
ssc00770	Pantothenate and CoA biosynthesis—Sus scrofa (pig)	0.01205545	1.918817	*UPB1//VNN1*
ssc04152	AMPK signaling pathway—Sus scrofa (pig)	0.02220957	1.65346	*ADIPOQ//ADIPOR2//FASN//SLC2A4*
ssc04976	Bile secretion—Sus scrofa (pig)	0.02225764	1.652521	*ABCB11//ADCY4//ADCY6*
ssc04920	Adipocytokine signaling pathway—Sus scrofa (pig)	0.0249136	1.603563	*ADIPOQ//ADIPOR2//SLC2A4*
ssc00983	Drug metabolism—other enzymes—Sus scrofa (pig)	0.03062382	1.513941	*CES1//UPB1*
ssc04910	Insulin signaling pathway—Sus scrofa (pig)	0.0323184	1.49055	*FASN//PPP1R3D//SLC2A4//SOCS2*
ssc05143	African trypanosomiasis—Sus scrofa (pig)	0.03438215	1.463667	*IDO2//LOC396781*
ssc05414	Dilated cardiomyopathy (DCM)—Sus scrofa (pig)	0.0360517	1.443074	*ADCY4//ADCY6//LOC396781*
ssc05340	Primary immunodeficiency—Sus scrofa (pig)	0.03632543	1.439789	*IL2RG//LOC396781*
ssc04911	Insulin secretion—Sus scrofa (pig)	0.03717241	1.429779	*ABCC8//ADCY4//ADCY6*
ssc04727	GABAergic synapse—Sus scrofa (pig)	0.03831094	1.416677	*ADCY4//ADCY6//GABBR1*
ssc04072	Phospholipase D signaling pathway—Sus scrofa (pig)	0.0386556	1.412788	*ADCY4//ADCY6//LOC396781//PTK2B*
ssc04974	Protein digestion and absorption—Sus scrofa (pig)	0.04064128	1.391033	*ACE2//COL7A1//SLC7A8*
ssc04912	GnRH signaling pathway—Sus scrofa (pig)	0.04304219	1.366106	*ADCY4//ADCY6//PTK2B*
ssc04914	Progesterone-mediated oocyte maturation—Sus scrofa (pig)	0.04304219	1.366106	*ADCY4//ADCY6//CPEB4*
ssc05032	Morphine addiction—Sus scrofa (pig)	0.04551314	1.341863	*ADCY4//ADCY6//GABBR1*
ssc01522	Endocrine resistance—Sus scrofa (pig)	0.0467747	1.329989	*ABCB11//ADCY4//ADCY6*

**Table 10:** The pathways with down-regulated gene enrichment scores.

Pathway ID	Definition	Fisher-P value	Enrichment_Score	Genes
ssc04918	Thyroid hormone synthesis—Sus scrofa (pig)	0.002669959	2.573495	*ATF4//TG//TTR*
ssc05030	Cocaine addiction—Sus scrofa (pig)	0.01468413	1.833152	*ATF4//PDYN*
ssc00980	Metabolism of xenobiotics by cytochrome P450—Sus scrofa (pig)	0.01528523	1.815728	*CYP2E1//EPHX1*
ssc05134	Legionellosis—Sus scrofa (pig)	0.01844662	1.734083	*CD14//TLR5*
ssc00561	Glycerolipid metabolism—Sus scrofa (pig)	0.02257206	1.646429	*AKR1B1//PNLIP*
ssc05204	Chemical carcinogenesis—Sus scrofa (pig)	0.02329364	1.632763	*CYP2E1//EPHX1*
ssc05031	Amphetamine addiction—Sus scrofa (pig)	0.02627419	1.580471	*ATF4//PDYN*
ssc05221	Acute myeloid leukemia—Sus scrofa (pig)	0.02627419	1.580471	*CD14//LEF1*
ssc04520	Adherens junction—Sus scrofa (pig)	0.03020581	1.519909	*LEF1//SSX2IP*
ssc05132	Salmonella infection—Sus scrofa (pig)	0.03961531	1.402137	*CD14//TLR5*
ssc04974	Protein digestion and absorption—Sus scrofa (pig)	0.04516019	1.345244	*PGA5//SLC7A9*
ssc04640	Hematopoietic cell lineage—Sus scrofa (pig)	0.04706952	1.32726	*CD14//GYPA*

### PCR validation of DEGs

**Figure 7:**
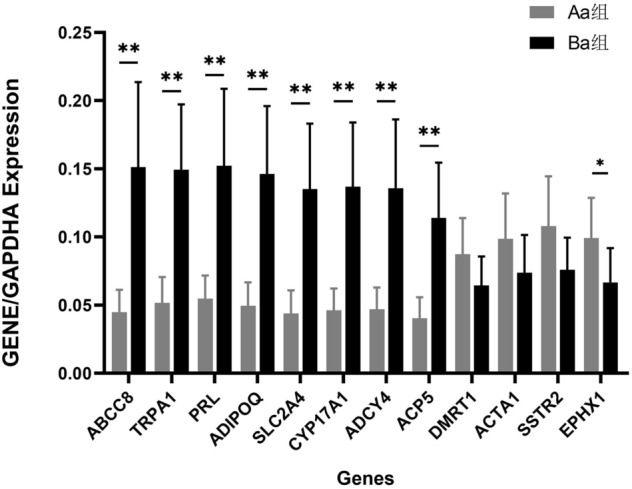
The selected 12 DEGs were validated by RT‐PCR. Error bars indicated mean ± standard errors of the mean. (*P < 0.05).

According to the microarray analysis results, 12 DEGs were selected for validation by real-time quantitative PCR. The selected DEGs are involved in nitric oxide metabolic process (PRL and ACP5), male gonad development (DMRT1), muscle fiber development (ACTA1), transition metal ion binding and metal ion binding (CYP17A1 and ACP5), integral component of the plasma membrane (SSTR2), endoplasmic reticulum membrane (EPHX1), type II diabetes mellitus pathway (ABCC8, ADIPOQ, and SLC2A4), inflammatory mediator regulation of TRP channels pathway (ADCY4 and TRPA1), and AMPK signaling pathway (ADIPOQ and SLC2A4). The results are shown in Table 11 and Fig. 7. The results indicate that the microarray data accurately reflects the gene expression patterns.

**Table 11:** The expression profiles of selected genes from microarray and RT-PCR.

Genbank accession	Gene symbol	Gene name	Description	Fold change by microarrays	Fold change by RT-PCR
CD572197	**ABCC8**	ATP binding cassette subfamily C member 8	ATP binding cassette subfamily C member 8 [Source:HGNC Symbol;Acc:HGNC:59] [ENSSSCT00000014612]	2.332	3.370**
XM_001926115	**TRPA1**	Transient receptor potential cation channel subfamily A member 1	PREDICTED: Sus scrofa transient receptor potential cation channel, subfamily A, member 1 (TRPA1), mRNA [XM_001926115]	2.104	2.887**
NM_213926	**PRL**	Prolactin	Sus scrofa prolactin (PRL), mRNA [NM_213926]	2.040	2.775**
JX092267	**ADIPOQ**	Adiponectin, C1Q and collagen domain containing	Rep: Adiponectin—Sus scrofa (Pig), complete [TC535135]	2.667	2.955**
AB005285	**SLC2A4**	Solute carrier family 2 member 4	GB	2.520	3.091**
NM_214428	**CYP17A1**	Cytochrome P450 17A1	Sus scrofa cytochrome P450 17A1 (CYP17A1), mRNA [NM_214428]	2.059	2.962**
XM_013978180	**ADCY4**	Adenylate cyclase 4	adenylate cyclase 4 [Source:HGNC Symbol;Acc:HGNC:235] [ENSSSCT00000002224]	2.443	2.886**
NM_214209	**ACP5**	Acid phosphatase 5, tartrate resistant	Sus scrofa acid phosphatase 5, tartrate resistant (ACP5), mRNA [NM_214209]	2.459	2.816**
NM_214111	**DMRT1**	Doublesex and mab-3 related transcription factor 1	Sus scrofa doublesex and mab-3 related transcription factor 1 (DMRT1), mRNA [NM_214111]	− 2.050	− 1.356
NM_001167795	**ACTA1**	Actin, alpha 1, skeletal muscle	Sus scrofa actin, alpha 1, skeletal muscle (ACTA1), mRNA [NM_001167795]	− 2.139	− 1.337
NM_001011694	**SSTR2**	Somatostatin receptor 2	Sus scrofa somatostatin receptor 2 (SSTR2), mRNA [NM_001011694]	− 2.130	− 1.420
NM_214355	**EPHX1**	Epoxide hydrolase 1	Sus scrofa epoxide hydrolase 1 (EPHX1), mRNA [NM_214355]	− 2.082	− 1.488*

Positive number indicates elevated expression (fold) in the meniscus of the experimental group (Ba group) compared to the meniscus of the control group (Aa group). Negative number indicates decreased expression (fold) in the meniscus of the experimental group (Ba group) compared to the meniscus of the control group (Aa group).

*p < 0.05, **p < 0.01 (versus sham-operated controls).

## Discussion

The insufficiency of the ACL can lead to meniscal damage^16^. A previous study of meniscal degeneration models in mini-pigs showed that removing the ACL leads to meniscal degeneration changes as observed by histology^11^. In addition, the real-time quantitative PCR analysis showed that COL1A1 is highly expressed in the degenerative meniscus^11^. In this study, the meniscus degeneration model was constructed by removing the ACL and LCL, based on the Pond-Nuki model^10^. The function of the meniscus is mainly to increase knee stability, absorb shocks, and conduct loads. When other stabilizing structures of the knee joint (such as the ligaments) are impaired, the contribution of the meniscus is increased, leading to stress and wear. This stress is mainly manifested in the horizontal shear plane and is the main cause of meniscal tear after ACL rupture. In addition, after LCL resection, the knee joint shows a loose lateral stable structure, causing varus change of the knee and shifting the rear limb force line inward. More stress is then taken by the medial meniscus, which can reach 60–80% of the BW^17^. The advantage of the mini-pig model over smaller animals is that the degree of the load is close to that of the human characteristics, but the disadvantages include longer modeling time and higher animal facility requirements^5^.

The results of the DEG cluster analysis showed that all samples could be accurately aggregated into the right grouping, indicating that the experimental intervention induced the DEGs. This is consistent with other studies that showed that ACL resection induces morphological, histological, and genetic changes in the meniscus^11,18^. A study in humans showed that changes in DEGs occur in ligaments after injury and that such changes might be observed in other musculoskeletal tissues^19^.

Meniscal stress is associated with inflammation. One of the most important mediators in inflammation is nitric oxide, wherein it upregulates meniscus matrix catabolism and pro-inflammatory gene expression, resulting in increased meniscus glycosaminoglycan release and meniscal impairment^20,21^. Nitric oxide can reduce autophagy by down-regulating the JNK signaling pathway, thereby affecting meniscal repair and causing meniscal degeneration^22^. Hyaluronic acid, selenium, and interleukin-10 delay or improve meniscus degeneration due to the inhibition of nitric oxide^23–25^. This study showed that the upregulated biological processes included regulation of nitric oxide biosynthesis process, nitric oxide biosynthetic process, and nitric oxide metabolic process, indicating that nitric oxide plays an important role in meniscal degeneration.

There is a clear sex difference in knee osteoarthritis and meniscus degeneration^26–28^, suggesting the role of sex hormones in meniscal degeneration. Although our study used male Wuzhishan mini-pigs, many male-associated biological processes were down-regulated in the DEG analysis, such as sex determination, negative regulation of the reproductive process, male gonadal development, primary male sexual characteristics development, male sex differentiation, and reproductive structure development. This suggests that the main feature of male characteristics are down-regulated by meniscal degeneration, indicating that androgens might play a role in promoting meniscus damage repair, while its decreased expression level promotes meniscal degeneration. Secondly, sex hormones might indirectly cause meniscal degeneration by affecting muscle strength, walking endurance, and balance ability^29^. Indeed, androgens promote the development of the skeletal muscle system^30,31^. When this dynamic stabilization mechanism decreases, abnormal stress on the knee meniscus increases, causing meniscal degeneration through nitric oxide, as discussed above.

Calcification is common in meniscal degeneration^32–34^. Mechanical stress and inflammatory factors promote the overexpression of ANKH and ENPP1 in meniscal fibrochondrocytes, leading to increased intracellular calcium concentration or enhanced calcium signaling that affects the cell function, impairing the meniscal repair capacity^35–37^. Phosphocitrate can inhibit the proliferation of meniscal cells and calcification, delaying and reversing meniscal degeneration and osteoarthritis^38^. In this study, upregulated transition metal ion binding was observed, and calcium is a transition metal. Upregulated iron ion binding was also observed, and mice with hereditary hemochromatosis are more prone to knee osteoarthritis and meniscal degeneration due to iron overload^39^.

In the pathway analysis, this study found that the upregulated pathways with the highest enrichment scores were type II diabetes mellitus, inflammatory mediator regulation of TRP channels, and AMPK signaling pathway. Insulin-like growth factors (IGFs) play an extremely important role in normal physiological activities, including musculoskeletal system development and meniscal repair^40^. Impaired insulin secretion leads to declination in the use of glucose by the cells, which in turn stimulates the AMPK signaling pathway, leading to enhanced catabolism and inhibited anabolism. The incidence of meniscal degeneration or osteoarthritis in patients with type II diabetes mellitus is higher than in normal individuals^41^. Damaged joints cause a series of reactions due to pro-inflammatory factors (such as IL-1 and TNF-α) and activate the MAPK pathways, such as increased MMPs secretion, ADAMTS-4 enhanced activity, increased chemokine concentration, and increased nitric oxide synthesis, among others^21,42,43^.

Those results are supported by a study in humans that showed that meniscal degeneration involves the biological processes of immune response, inflammatory response, biomineral formation, and cell proliferation^44^. Despite the differences between humans and mini-pigs, the results of the involved pathways and genes are consistent between the two species.

There are some limitations to the present study. First, the experiment used the contralateral leg as a control group. The biomechanical characteristics of the contralateral leg also change during walking because of the affected leg, which in turn can cause some changes in gene expression. Therefore, it cannot be considered an ideal control group. Second, meniscal tissue is composed of a variety of cells; therefore, the DEGs are, in fact, the response of the overall tissue to modeling. The role of each cell constituent of the meniscus in degeneration is difficult to define clearly. Further research should be conducted through cell isolation culture or single-cell gene sequencing^44,45^. Third, the results obtained from animal experiments might differ from humans, suggesting only possible mechanisms for human meniscal degeneration.

## Conclusion

Wuzhishan mini-pigs can be used as animal models for studying meniscus degeneration through the resection of the ACL and LCL. The present study provides some insight into the molecular mechanisms underlying meniscal degeneration. They might also reveal potential targets for meniscal degeneration treatment and early diagnosis.

## Data Availability

The datasets used and/or analyzed during the current study are available from the corresponding author on reasonable request.
